# The Incidence of Epstein-Barr Virus-Positive Diffuse Large B-Cell Lymphoma: A Systematic Review and Meta-Analysis

**DOI:** 10.3390/cancers13081785

**Published:** 2021-04-08

**Authors:** Jisun Hwang, Chong Hyun Suh, Kyung Won Kim, Ho Sung Kim, Philippe Armand, Raymond Y. Huang, Jeffrey P. Guenette

**Affiliations:** 1 Department of Radiology, Dongtan Sacred Heart Hospital, Hallym University Medical Center, 7, Keunjaebong-gil, Hwaseong-si, Gyeonggi-do 18450, Korea; biydjs@hallym.or.kr; 2 Asan Medical Center, Department of Radiology and Research Institute of Radiology, University of Ulsan College of Medicine, Seoul 05505, Korea; kyungwon_kim@amc.seoul.kr (K.W.K.); radhskim@gmail.com (H.S.K.); 3 Department of Medical Oncology, Dana-Farber Cancer Institute, Harvard Medical School, Boston, MA 02115, USA; Philippe_Armand@dfci.harvard.edu; 4 Division of Neuroradiology, Brigham and Women’s Hospital, Dana-Farber Cancer Institute, Harvard Medical School, Boston, MA 02115, USA; ryhuang@bwh.harvard.edu (R.Y.H.); jpguenette@bwh.harvard.edu (J.P.G.)

**Keywords:** in situ hybridization, meta-analysis, systematic review, lymphoma, immunohistochemistry

## Abstract

**Simple Summary:**

The worldwide prevalence of Epstein-Barr virus-positive (EBV+) diffuse large B-cell lymphoma (DLBCL) is undetermined. There is no clearly defined cut-off for EBV-encoded RNA (EBER) positivity in tumor cells by in situ hybridization. A lack of common criteria for positive expression of EBER has been raised as a limitation for interpreting and understanding the geographic and ethnic disparity of prevalence of EBV+ DLBCL. We conducted a systematic literature review and meta-analysis to establish the proportions of EBV+ DLBCL patients. Results showed that the pooled proportion of EBER positivity was 7.9% in patients with de novo DLBCL. The prevalence of EBV+ DLBCL was significantly higher in Asia and South America compared with Western countries. A tendency for lower pooled proportions was observed in studies using a higher cut-off for EBER positivity. The patients’ age did not significantly affect the prevalence. These findings may improve our current knowledge of the EBV+ DLBCL.

**Abstract:**

The worldwide prevalence of Epstein-Barr virus-positive (EBV+) diffuse large B-cell lymphoma (DLBCL) is undetermined. There is no clearly defined cut-off for EBV-encoded RNA (EBER) positivity in tumor cells by in-situ hybridization. The purpose of this study was to establish the proportions of EBV+ DLBCL patients and influence of the different cut-offs for EBER positivity, geographical location, and age on the prevalence of EBV+ DLBCL. PubMed and EMBASE were searched for studies published up to May 28, 2020 that reported proportions of EBER positivity in immunocompetent and de novo DLBCL patients. The pooled proportions were computed by an inverse variance method for calculating the weights and the DerSimonian–Laird method. Multiple subgroup analyses were conducted to explore any heterogeneity. Thirty-one studies (8249 patients) were included. The pooled proportion of EBV+ DLBCL was 7.9% (95% CI, 6.2–10.0%) with significant heterogeneity among studies (*p* < 0.001). The prevalence of EBV+ DLBCL was significantly higher in Asia and South America compared with Western countries (*p* < 0.01). The cut-offs for EBER positivity (10%, 20%, 50%) and patients’ age (≥50 years vs. <50 years) did not significantly affect the prevalence (*p* ≥ 0.10). EBV+ DLBCL is rare with a pooled proportion of 7.9% in patients with DLBCL and the geographic heterogeneity was confirmed.

## 1. Introduction

Epstein-Barr virus (EBV) infection is common and affects the majority of individuals worldwide [1]. The primary infection usually takes place during childhood and then the virus undergoes a latency phase without causing any symptoms [1]. However, in some individuals, the virus is associated with a wide range of lymphoid malignancies, such as Burkitt’s lymphoma, B-cell lymphoproliferative diseases, and Hodgkin lymphoma [1]. The concept of EBV-associated B-cell lymphoproliferative disorders without a predisposing immunodeficiency condition was first described in two studies by Oyama et al., in which the patients tended to be older and in which the disease was associated with aggressive clinical features with relatively poor clinical outcomes in response to conventional chemotherapy [2,3]. As a result, ‘EBV-positive (EBV+) diffuse large B cell lymphoma (DLBCL) of the elderly’ was incorporated as a new subtype of DLBCL in the 4th edition of the 2008 World Health Organization (WHO) Classification of Tumours of Haematopoietic and Lymphoid Tissues [4]. Subsequently, evidence for EBV+ DLBCL in younger patients was found in several studies and the terminology has been changed to ‘EBV+ DLBCL, not otherwise specified’ without an age restriction in the 2016 WHO classification [5,6,7].

Detection of the EBV-encoded RNA (EBER) in the nuclei of tumor cells by in situ hybridization is the gold standard method for the evaluation of EBV+ DLBCL. However, there has been no clearly defined cut-off for EBER positivity, even in the 2008 and 2016 WHO classifications, and various cut-offs have been adopted in previous studies, from 5% to 80% [8,9,10]. The general prevalence of EBV+ DLBCL remains undetermined due to many factors, such as the scarcity of large-scale studies, the lack of consensus regarding the optimal cut-off for EBER positivity, and the geographic variation in the prevalence [11]. The reported incidence of EBV+ DLBCL has varied with a tendency towards a high prevalence (up to 28%) in Asia and South America, whereas a low prevalence has been reported in Western studies [9,12,13,14]. In this context, this systematic review and meta-analysis was designed to develop more comprehensive estimates of the prevalence of EBV+ DLBCL from the worldwide literature to date. Subgroup analysis was also performed to explore the influence of the cut-off for EBER positivity, geographical location, and age on the prevalence of EBV+ DLBCL.

## 2. Methods

### 2.1. Search Strategy and Selection Criteria

Studies were identified by searching the electronic databases of PubMed and EMBASE, which were published up to 28 May 2020. We used the following search terms: ((Diffuse large B-cell lymphoma) OR (DLBCL)) AND ((Epstein–Barr virus) OR (EBV-associated) OR (EBV+) OR (EBV positive)) AND ((EBV-encoded RNA) OR (EBER) OR (immunohistochemistry) OR (prevalence) OR (incidence)). The bibliographies of the retrieved studies were thoroughly checked for the identification of any other relevant studies. Article searches were restricted to the English language literature.

The inclusion criteria were as follows: (1) histopathological diagnosis of de novo or newly diagnosed DLBCL according to the WHO classification; (2) detailed data sufficient to evaluate the proportion of EBER positivity (i.e., EBV+ DLBCL) by in situ hybridization; and (3) exclusion of immunodeficiency state. The exclusion criteria were: (1) insufficient raw data for estimating the outcome; (2) review or opinion; (3) case reports or series having 10 cases or fewer; and (4) multiple studies with overlapping study samples. The studies with a larger number of patients were selected when overlapping study samples were identified. The study selection process was independently performed by two reviewers (J.H. and C.H.S.) and disagreements were resolved by consensus.

### 2.2. Data Extraction and Quality Assessment

Information was retrieved from each included study on: (1) characteristics of the study (author, study period, institution, design); (2) characteristics of the patients (country, geographical location, age, sex, clinical setting, international prognostic index, Ann Arbor Stage, elevated LDH, front-line treatment); (3) pathological characteristics (cut-off for a positive expression of EBER, EBER status).

Quality assessment of the included studies was evaluated using the Newcastle-Ottawa scale for cohort and case-control studies [15,16]. The Newcastle-Ottawa scale comprises three domains of Selection, Comparability, and Outcome (or Exposure in case-control studies). A study can be assigned one score in each item for the Selection and Outcome domains and two scores for the Comparability domain. The quality of a study was judged by a total score: 8–9, very good; 6–7, good; 4–5, satisfactory; 0–3, unsatisfactory. Two reviewers (J.H. and C.H.S.) independently extracted the data and conducted the quality assessment, and disagreements were resolved by consensus.

### 2.3. Data Synthesis and Analysis

The primary outcome measure was the pooled proportion of EBER positivity by in situ hybridization among DLBCL patients. The secondary outcomes was subgroup analysis for the studies according to the cut-offs for a positive expression of EBER, geographical location, age (elderly ≥50 years vs. young <50 years) [4]. When sufficient data for calculating the proportion of EBV+ DLBCL for each elderly or young patient was given in the included studies, all of the outcomes (i.e., EBER positivity among the total patients, elderly patients, and young patients) were extracted. In studies with multiple cut-offs for EBER positivity, the data from each lowest and highest threshold was used for calculating the pooled proportion separately.

The pooled proportions of EBV+ DLBCL were computed by the inverse variance method for calculating the weights and the DerSimonian–Laird method [17]. The preset cut-offs in the individual studies were used for the analysis. The Q test or the inconsistency index (I^2^) statistic was used to assess statistical heterogeneity across studies, and *p* < 0.1 on the Q test and I^2^ ≥ 50% were used to define significant heterogeneity [18]. Publication bias was evaluated using the funnel plot and Egger’s test [19]. Multiple subgroup analyses were examined according to the cut-off for a positive expression of EBER, geographical location, and age.

Statistical analysis was performed by one author (C.H.S.) with the “meta” package in R software version 4.0.2 (R Foundation for Statistical Computing).

## 3. Results

### 3.1. Study Search and Quality Assessment

**Figure 1:**
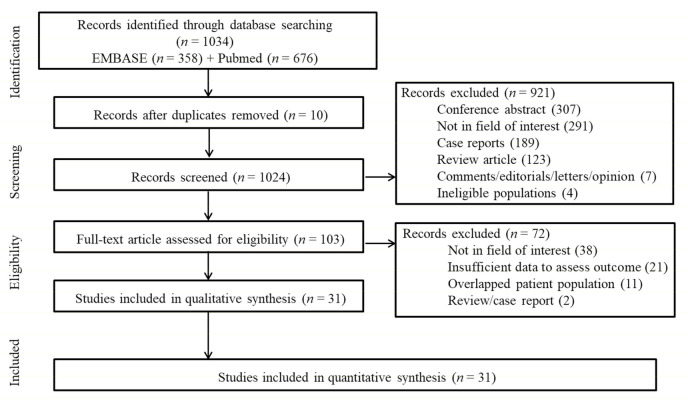
Flow diagram of the included studies.

A total of 1034 studies were identified by the literature search. After adjusting for duplicates, 1024 articles remained. Of these, 921 articles were removed after reviewing the titles and abstracts (Figure 1). After full-text scrutiny of the remaining 103 articles, 72 studies were further excluded due to the following criteria: 38 studies were not in the field of interest, 21 studies had insufficient data to evaluate the outcome, 11 studies included a partially overlapping patient population, and one each were a review and a case report. Finally, 31 studies comprised of 8249 patients were included in the meta-analysis [5,6,8,9,10,12,13,20,21,22,23,24,25,26,27,28,29,30,31,32,33,34,35,36,37,38,39,40,41,42,43].

Twenty studies were classified as having a very good quality, eight studies as fair quality, and three studies as satisfactory quality (Supplementary Table S1). Because the Newcastle-Ottawa scale was developed for cohort and case-control studies, we assigned the Selection domain for a secondary analysis study of previous clinical trials as good quality. In the Selection domain, most of the included studies were of good quality, except for one study, which derived the subjects from a reference laboratory population [29]. In ten out of the 31 studies, no explicit mention of differences in baseline characteristics was documented for the assessment of the Comparability domain. In nine studies, the follow-up duration was not mentioned, or the follow-up rate was inadequate (<80%), or both, in the assessment of the Outcome domain.

### 3.2. Study Characteristics

The study and patient characteristics of the 31 included studies are presented in Table 1 and Table 2, respectively. In brief, the study design was retrospective in 18 studies, one was a secondary analysis of a primary clinical trial, and not-explained in the remaining 12 studies. Concerning the preset cut-offs for EBER positivity by in situ hybridization, various cut-offs were used among the included studies. One study used >5% [10], seven studies used >10% [9,20,21,23,27,28,29], seven studies used >20% [5,6,12,24,31,32,43], one study used >30% [35], three studies used >50% [33,38,39], and two studies used >80% [8,30]. Three studies evaluated the outcomes by two or more cut-offs among 10%, 20%, 30%, and 50% for EBER positivity [9,13,42]. Three studies investigated only extra-nodal DLBCL (primary gastrointestinal tract, sinonasal, and nasopharyngeal DLBCL). Fifteen studies were conducted in East Asian countries [5,8,13,20,22,23,26,27,30,31,33,35,37,39,42], five in the Middle East [6,21,32,40,41], three in Europe [10,34,38], four in North America [25,29,36,43], and two in South America [12,24]. A study by Gibson et al. considered EBV + DLBCL of the elderly for patients age 60 years or greater [25]. Except for this study, all of the other 12 studies that were performed for EBV + DLBCL of the elderly used an age cut-off of 50 years [5,6,10,13,20,23,32,33,37,38,39,41]. In 17 studies, all age groups were included with or without children [1,8,9,12,21,22,26,27,28,29,30,31,34,35,36,42,43].

### 3.3. The Pooled Proportion of EBER Positivity and Subgroup Analysis

**Figure 2:**
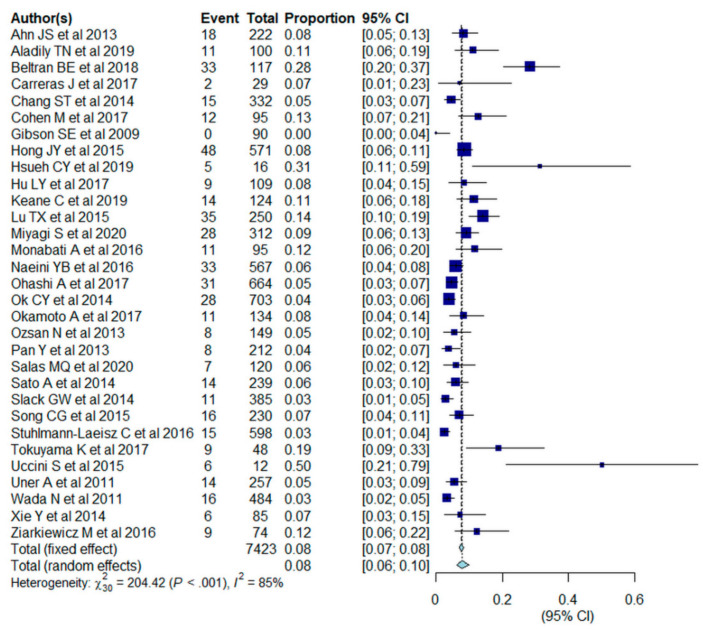
Forest plots of the pooled proportion of Epstein-Barr virus-positive (EBV+) diffuse large B cell lymphoma (DLBCL) in the included studies (*n* = 31).

**Figure 3:**
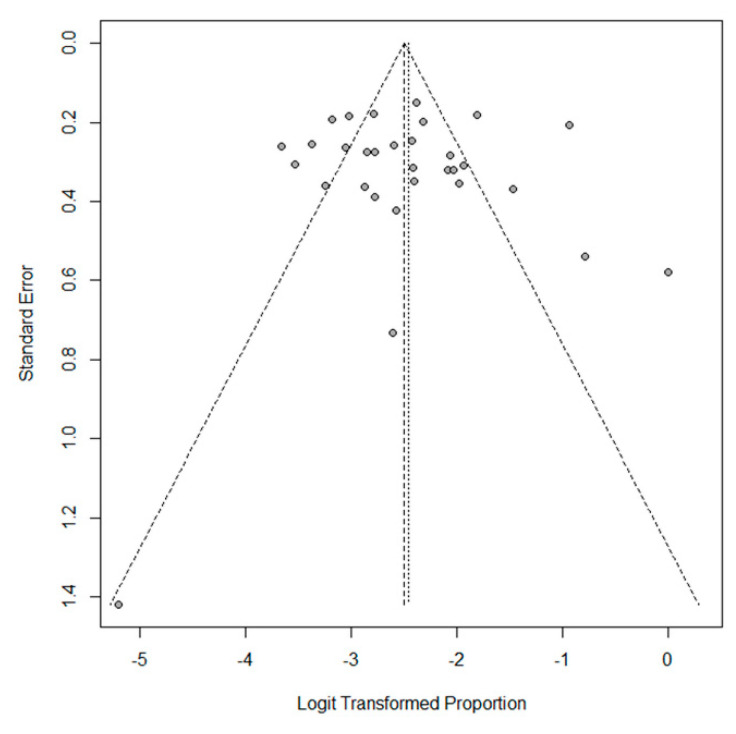
Funnel plot of the proportion of EBV+ DLBCL in the included studies. Note the absence of funnel plot asymmetry.

The pooled outcomes of the 31 included studies are summarized in Table 3. The proportion of EBV+ DLBCL ranged from 0 to 50.0%. The pooled proportion of EBV+ DLBCL was 7.9% (95% CI, 6.2–10.0%), with significant heterogeneity among studies (I^2^ = 85%, *p* < 0.001) (Figure 2). The funnel plot and Egger’s test (*p* = 0.77) suggested an absence of publication bias (Figure 3). The pooled proportion was 7.5 % (95% CI, 5.8–9.6%) when a data with highest threshold was chosen in studies with multiple cut-offs for EBER positivity (I^2^ = 86%, *p* < 0.001), which was similar to the proportion obtained with the lowest threshold (7.9%).

**Figure 4:**
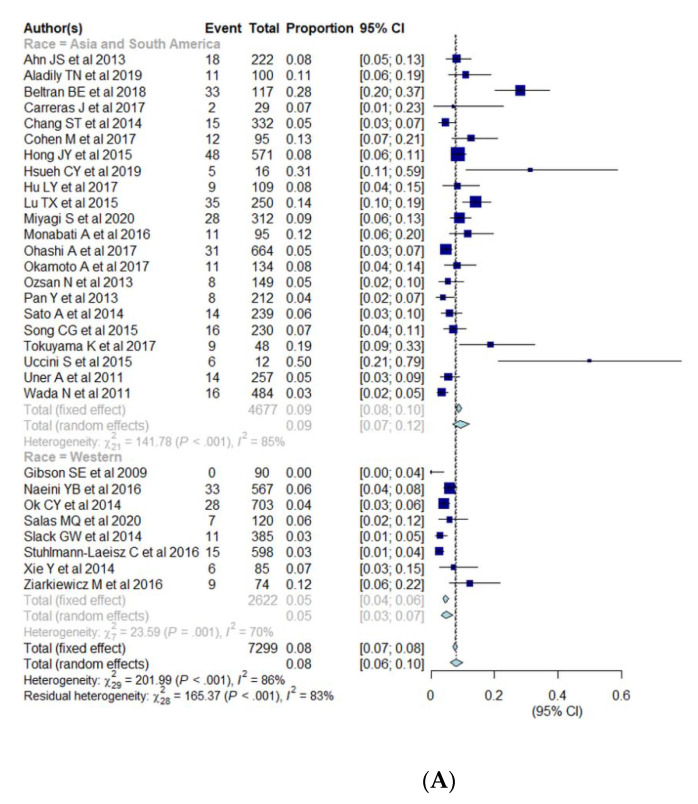

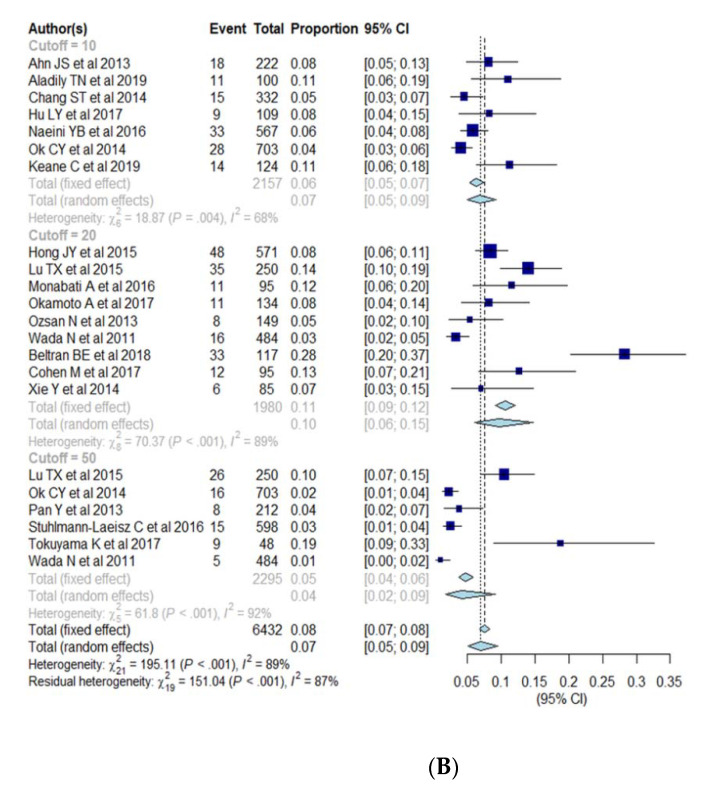

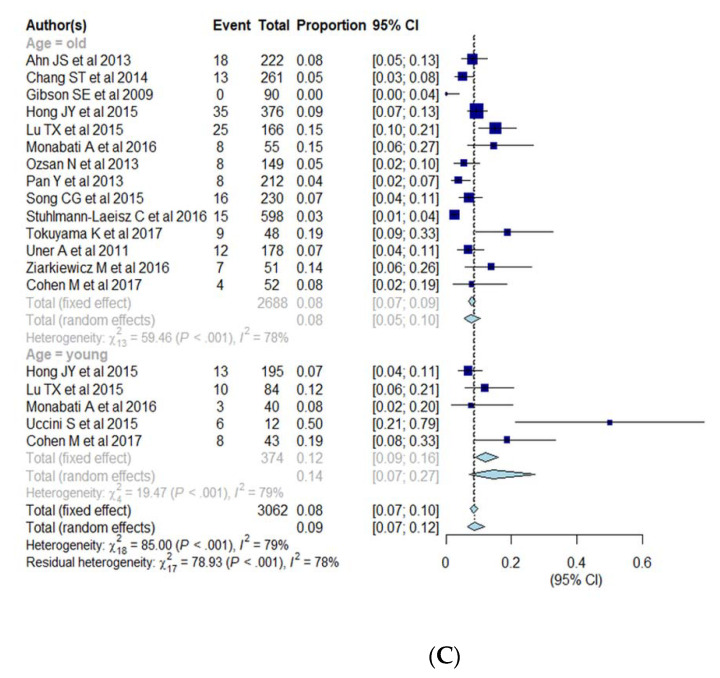
(**A**–**C**) Forest plots of the pooled proportions of EBER positive DLBCL. Subgroup analyses were according to race (Asia and South America vs. Western countries) (**A**), cut-offs for EBER positivity (10%, 20%, 50%) (**B**), and patients’ age (elderly ≥50 years vs. young <50 years) (**C**).

Subgroup analysis was performed for cut-offs for a positive expression of EBER (10%, 20%, 50%), race (Asia and South America vs. Western), age (elderly vs. young) (Table 3, Figure 4A–C). The pooled proportion of EBV+ DLBCL was significantly higher in Asia and South America (9.2%; 95% CI, 7.0–12.0%) compared with Western countries (4.7%; 95% CI, 3.2–6.8%) (*p* = 0.005). The pooled proportion of EBV+ DLBCL with cut-offs of >10%, >20%, and >50% were 6.8% (95% CI, 5.0–9.3%), 9.6% (95% CI, 6.1–14.8%), and 4.2% (95% CI, 1.9–9.2%), respectively. The pooled proportion of EBV+ DLBCL in the elderly and young patients were 7.6% (95% CI, 5.4–10.4%) and 14.4% (95% CI, 7.1–27.1%), respectively. The cutoffs for EBER positivity and patients’ age were not significant factors of heterogeneity with *p* values being 0.17 and 0.10, respectively. All of the variables (race, cutoffs for EBER positivity, age) show significant heterogeneity among studies (I^2^ > 50%, *p* < 0.01).

## 4. Discussion

In this systematic review and meta-analysis, we identified a pooled prevalence of 7.9% of EBER positivity among 8249 patients with de novo DLBCL. The prevalence of EBV+ DLBCL was significantly higher in Asia and South America (9.2%) compared with that in Western countries (4.7%; *p* <0.01). The cut-offs for EBER positivity (10%, 20%, 50% of tumor cells) and patients’ age (≥50 years vs. <50 years) did not significantly affect the prevalence of EBV+ DLBCL.

A geographic variation of EBV+ DLBCL has well been documented in previous studies, with relatively higher prevalence in Asia and South America than in Western countries [9,12,13,14,44,45,46]. Our study meta-analytic confirmed the geographic heterogeneity of the prevalence, which is consistent with previous studies. This result is similar to other EBV-associated disorders, which are more common in Asian and Latin American populations [33,45]. Likewise, a geographic difference in EBV strains has been proposed as a possible factor for the variations in the prevalence and clinical behavior of EBV+ DLBCL [9]. The composition of cut-offs for EBER positivity was not substantially different between Asian and Latin American, and Western studies. Approximately one half of each group (59% in Asian and Latin American and 43% in Western) used lower (10%, 20%, or 30%) cut-offs and a small percentage (18% and 14%) of each group used higher cut-offs (50% or 80%) for EBER positivity. Therefore, the geographic difference noted in this study might be less likely to be affected by the various cut-offs in the included studies. Recently, the EBV seropositive rate in children has decreased and the age of patients with primary EBV infection has increased in Korea and Japan [47,48]. It will remain to be seen whether the delay in the age of primary infection will affect the incidence of EBV-associated disorders in the Asian countries.

A lack of common criteria for positive expression of EBER has been raised as a limitation for interpreting and understanding the geographic and ethnic disparity of prevalence of EBV+ DLBCL. The included studies used a wide range of cut-offs from 5% to 80%. The most commonly used criteria were 10% and 20%. We expected that a lower cut-off for EBER positivity by in situ hybridization might be associated with a higher proportion of EBV+ DLBCL, as was noted in the previous studies [9,10,13,42]. Our meta-analysis showed a tendency for lower pooled proportions in studies using a cut-off of 50% compared to those of 10% or 20%. However, this finding did not reach statistical significance.

The EBV positivity was associated with a worse prognosis in DLBCL patients treated with chemotherapy [3,14]. After the introduction of rituximab, although various prognostic effects of EBV positivity have been reported [9,12,13,31], patients with EBV+ DLBCL still seem to have less favorable clinical outcomes compared with EBV-negative patients [11]. A recent meta-analysis demonstrated that EBV+ DLBCL was significantly associated with worse overall survival and progression-free survival [49]. Considering the trend in the prevalence according to different cutoffs in our study, it might be necessary to establish the most acceptable threshold for EBER positivity for better discrimination of patients at risk for worse survival. According to a study by Lu et al., although the prevalence of EBV+ DLBCL was lower when a higher cut-off for EBER positivity was used, patients with EBV+ DLBCL showed inferior prognosis compared with EBER-negative patients regardless of the cut-offs [13]. More research is needed regarding relationship between cut-offs for EBER positivity and prognosis of EBV+ DLBCL.

The age limit (over the age of 50) for a diagnosis of EBV+ DLBCL was eliminated in the revision of the 2016 WHO Classification [7]. The prevalence of EBV+ DLBCL in the studies published before the revision of WHO classification might be influenced by the age limit for diagnosis. We summarized the studies published both before the after the revision and found that the pooled proportion of EBV+ DLBCL was similar between the young and elderly patient groups. This finding is inconsistent with previous studies, because EBV+ DLBCL tends to be diagnosed in patients at an older age, although it has also been detected (less commonly) in younger patients [5,6,11]. Our study supports the fact that EBV+ DLBCL could be encountered in patients regardless of age. However, the results have to be interpreted carefully because of the relatively small number of studies in the subgroup of younger patients.

Our study has a limitation. The overall estimates in this study showed substantial statistical heterogeneity. Although we performed subgroup analysis, other unidentified factors might have been present, particularly for the unexpected results according to the cut-offs for EBER positivity and patients’ age.

## 5. Conclusions

This meta-analysis shows that EBV+ DLBCL is rare, with a pooled proportion of 7.9% among patients with de novo DLBCL. The geographic heterogeneity was confirmed with a higher prevalence in Asia and South America than in Western countries. There seems to be a trend of lower prevalence of EBV+ DLBCL in studies using a cut-off of 50% for EBER positive tumor cells. However, this finding did not reach statistical significance. The prevalence of EBV+ DLBCL was not influenced by the patients’ age.

## Figures and Tables

**Table 1:** Main characteristics of the included studies.

Authors, Publication Years	Patient Enrollment Period	Institution	Country	Design	EBER Cut-Off Values
Ahn JS et al., 2013 [20]	2003–2011	Chonnam National University Hwasun Hospital, Chonbuk National University Medical School	Korea	Retrospective	10
Aladily TN et al., 2019 [21]	NA	Jordan University Hospital, Necmettin Erbakan University	Jordan, Turkey	Retrospective	10
Beltran BE et al., 2018 [12]	2006–2015	Hospital Nacional Edgardo Rebagliati Martins	Peru	Retrospective	20
Carreras J et al., 2017 [22]	2002–2013	Tokai University, School of Medicine	Japan	NA	NA
Chang ST et al., 2014 [23]	1989–2010	Chi-Mei Medical Centre	Taiwan	Retrospective	10
Cohen M et al., 2017 [24]	1987–2013, 2009–2013	Ricardo Gutiérrez Children’s Hospital, National Academy of Medicine	Argentina	Retrospective	20
Gibson SE et al., 2009 [25]	2002–2008	Cleveland Clinic	USA	Retrospective	NA
Hong JY et al., 2015 [5]	1995–2011	Samsung Medical Center	Korea	Secondary analysis	20
Hsueh CY et al., 2019 [26]	1995–2017	Taipei Veterans General Hospital	Taiwan	Retrospective	NA
Hu LY et al., 2017 [27]	2005–2012	Sun Yat-sen University Cancer Center	China	Retrospective	10
Keane C et al., 2019 [28]	2003–2014	Multicenter	Australia	NA	10
Lu TX et al., 2015 [13]	2006–2014	Jiangsu Province Hospital	China	NA	20, 50
Miyagi S et al., 2020 [8]	1995–2018	Nagoya University Hospital	Japan	Retrospective	80
Monabati A et al., 2016 [6]	2012–2014	Shahid Fagihi hospital	Iran	Retrospective	20
Naeini YB et al., 2016 [29]	2008–2015	Clarient Pathology Services	USA	NA	10
Ohashi A et al., 2017 [30]	NA–2012	Fujita Health University School	Japan	NA	80
Ok CY et al., 2014 [9]	NA	Multicenter (the International DLBCL Rituximab-CHOP Consortium Program Study)	Western countries	NA	10, 30, 50
Okamoto A et al., 2017 [31]	2007–2012	Fujita Health University Hospital	Japan	Retrospective	20
Ozsan N et al., 2013 [32]	2009–2012	Ege University Faculty of Medicine	Turkey	Retrospective	20
Pan Y et al., 2013 [33]	1999–2010	Tianjin Medical University Cancer Institute and Hospital	China	NA	50
Salas MQ et al., 2020 [34]	2012–2016	Catalan Institue of Oncology	Spain	Retrospective	NA
Sato A et al., 2014 [35]	2007–2011	Tokai University Hospital	Japan	NA	30
Slack GW et al., 2014 [36]	1999–2016	British Columbia Cancer Agency	Canada	NA	NA
Song CG et al., 2015 [37]	2001–2011	Sun Yat-Sen University Cancer Center	China	Retrospective	NA
Stuhlmann-Laeisz C et al., 2016 [38]	NA	Multicenter	Switzerland, Germany	NA	50
Tokuyama K et al., 2017 [39]	2007–2016	Oita University Faculty of Medicine	Japan	Retrospective	50
Uccini S et al., 2015 [40]	2008–2013	Children’s Welfare Teaching Hospital	Iraq	NA	NA
Uner A et al., 2011 [41]	1999–2009	Hacettepe University and Gazi University Medical Schools	Turkey	Retrospective	NA
Wada N et al., 2011 [42]	1999–2009	Multicenter (Osaka Lymphoma Study Group)	Japan	NA	20, 50
Xie Y et al., 2014 [43]	2002–2012	University of Southern California Medical Center	USA	Retrospective	20
Ziarkiewicz M et al., 2016 [10]	1994–2011	Medical University of Warsaw	Poland	Retrospective	5

NA = not available; EBER = Epstein-Barr virus-encoded RNA.

**Table 2:** Patient characteristics of the included studies.

Authors	Patients (*N*)	Age (Range)	Male to Female Ratio	Clinical Setting	IPI Category	Ann Arbor Stage	Elevated LDH	Treatment Arm
Ahn JS et al., 2013 [20]	222	66 (51–82) in EBV+, 67 (50–86) in EBV−	131:91	DLBCL	0–1 (33%), 2 (20%), 3 (19%), 4–5 (24%)	3–4 (52%)	56.0%	R-CHOP
Aladily TN et al., 2019 [21]	100	NA	NA	DLBCL	NA	NA	NA	NA
Beltran BE et al., 2018 [12]	117	NA	NA	DLBCL	0–2 (40.2%), 3–5 (59.8%)	3–4 (43.6%)	51.3%	R-CHOP, CHOP
Carreras J et al., 2017 [22]	29	NA	20:9	Primary sinonasal DLBCL	0–2 (72.4%), 3–5 (20.7%)	1–2 (72.4%), 3–4 (27.6%)	34.5%	R-CHOP, R-CHOP-like
Chang ST et al., 2014 [23]	424	74 (41–91) in EBV+, 65 (14–94) in EBV−	194:138	DLBCL	NA	NA	NA	CEOP, CHOP, R-CEOP in EBV+
Cohen M et al., 2017 [24]	102	52 (2–84)	50:52	DLBCL	NA	3–4 (57%)	NA	GATLA treatment protocols (pediatric), R-CHOP (adult)
Gibson SE et al., 2009 [25]	90	NA	NA	DLBCL	NA	NA	NA	NA
Hong JY et al., 2015 [5]	571	55 (16–88)	335:236	DLBCL	0–2 (69.5), 4–5 (30.5%)	1–2 (57.7%), 3–4 (42.3%)	65.4%	R-CHOP, CHOP, others
Hsueh CY et al., 2019 [26]	17	61.59 (17–88)	9:8	Nasopharyngeal DLBCL	NA	1 (23.5%), 2 (47.1%), 4 (29.4%)	NA	NA
Hu LY et al., 2017 [27]	204	52 (18–86)	115:89	DLBCL	0–2 (77.9%), 3–5 (22.0%)	3–4 (51.0%)	48.4%	R-CHOP or R-CHO-like regimen, CHOP or CHOP-like regimen, MA regimen
Keane C et al., 2019 [28]	433	NA	192:241	DLBCL	0 (10.5%), 1–2 (45.0%), 3–5 (44.5%)	3–4 (52.9%)	44.1%	R-CHOP, alternative regimens
Lu TX et al., 2015 [13]	250	NA	144:106	DLBCL	0–2 (73.4%), 3–5 (26.6%)	1–2 (50%), 3–4 (50.0%)	45.6%	R-CHOP, R-DA-EPOCH, CHOP
Miyagi S et al., 2020 [8]	312	69.5 (35–84)	16:20	Primary gastrointestinal tract DLBCL	3–5 (28.6%) in EBV+	^a^ I (25%), II1-IV (75%) in EBV+	42.8% in EBV+	R-containing CTx in 25/28 (89.3%)
Monabati A et al., 2016 [6]	95	53.9 (12–90)	52:43	DLBCL	NA	NA	NA	NA
Naeini YB et al., 2016 [29]	677	67 (11–96)	378:285	DLBCL	NA	NA	NA	NA
Ohashi A et al., 2017 [30]	667	NA	385:278	DLBCL	4–5 (46.8)	3–4 (54.4%)	58.9%	NA
Ok CY et al., 2014 [9]	732	63 (16–95)	421:311	DLBCL	0–2 (59.3%), 3–5 (40.7%)	1–2 (46.4%), 3–4 (53.6%)	62.3%	R-CHOP
Okamoto A et al., 2017 [31]	134	77 (62–91) in EBV+, 38 (33–93) in EBV−	79:55	DLBCL	0–2 (47.8%), 3–5 (52.2%)	1–2 (42.5%), 3–4 (57.5%)	61.2%	Rituximab+anthracycline-based CTx in 93%
Ozsan N et al., 2013 [32]	149	>50	NA	DLBCL	NA	NA	NA	R-CHOP in EBV+
Pan Y et al., 2013 [33]	212	58.5 (22–91)	115:97	DLBCL	NA	NA	NA	R-CHOP, CHOP in EBV+
Salas MQ et al., 2020 [34]	216	63 (19–90)	105:111	DLBCL	1–2 (49.5%), 3–5 (50.5%)	1–2 (31.0%), 3–4 (68.9%)	56.5%	Various
Sato A et al., 2014 [35]	239	71.5 in EBV+, 68.0 in EBV−	130:109	DLBCL	3–5 (48.6%)	3–4 (53.5%)	61.1%	R-CHOP, R-CHOP-like
Slack GW et al., 2014 [36]	385	64 (16–92)	195:113	DLBCL	0–2 (35%), 3–5 (65%)	1–2 (46%), 3–4 (58%)	50.0%	R-CHOP
Song CG et al., 2015 [37]	230	62 (51–76)	147:83	DLBCL	0–2 (75.2%), 3–5 (24.8%)	1–2 (35.7%)	48.7%	CHOP or EPOCH +/− Rituximab
Stuhlmann-Laeisz C et al., 2016 [38]	598	70 (50–98)	NA	DLBCL	NA	NA	NA	CHOP, R-CHOP, R-Bendamustin in EBV+
Tokuyama K et al., 2017 [39]	48	76.2 (72–83) in EBV+, 71.3 (53–91) in EBV−	15:23	DLBCL	NA	1–2 (35.4%), 3–4 (64.6%)	NA	NA
Uccini S et al., 2015 [40]	13	8.8 in EBV+, 7.5 in EBV−	10:3	pediatric DLBCL	NA	^b^ 1 (7.7%), 2 (15.4%), 3 (53.8%), 4 (23.0%)	NA	Various
Uner A et al., 2011 [41]	340	54.5 (6–101)	170:170	DLBCL	NA	NA	NA	NA
Wada N et al., 2011 [42]	484	68 (16–95)	1.29:1	DLBCL	NA	NA	NA	NA
Xie Y et al., 2014 [43]	85	54 (20–89)	51:34	DLBCL	0–2 (65%), 3–5 (36%)	1 (21%), 2 (26%), 3 (12%), 4 (41%)	53.0%	R-CHOP, R-CHOP-like
Ziarkiewicz M et al., 2016 [10]	74	63.5 (23–86)	37:37	DLBCL	0–2 (58.6%)	0–2 (40.8%)	71.9%	CHOP, CHOP-variant, +/−Rituximab

^a^ Lugano staging system; ^b^ St. Jude’s staging classification for childhood non-Hodgkin lymphoma; NA = not available; IPI = international prognostic index; LDH = lactate dehydrogenase; R-CHOP = rituximab, cyclophosphamide, doxorubicin, vincristine, and prednisone.

**Table 3:** Summary of the meta-analytic pooled proportion for various outcomes among the included studies.

Outcome	No. of Studies	Summary Estimate			*p*-Value by Test for Subgroup Differences
		Pooled Proportion (%) (95% CI)	*p* Value for Heterogeneity	I^2^ (%)	
EBER positive	31	7.9 (6.2–10.0)	<0.001	85	–
Asia and South America	22	9.2 (7.0–12.0)	<0.001	83	0.005
Western	7	4.7 (3.2–6.8)	<0.001	74	
EBER positive (cut-off: 10%)	7	6.8 (5.0–9.3)	0.004	68	0.17
EBER positive (cut-off: 20%)	9	9.6 (6.1–14.8)	<0.001	89	
EBER positive (cut-off: 50%)	6	4.2 (1.9–9.2)	<0.001	92	
Elderly EBER positive	13	7.6 (5.4–10.4)	<0.001	78	0.10
Young EBER positive	5	14.4 (7.1–27.1)	<0.001	79	

## Data Availability

The databases for the analyses of this study are available on request from the corresponding author.

## Supplementary Materials

The following are available online at https://www.mdpi.com/article/10.3390/cancers13081785/s1, Table S1: Quality ratings for the included studies on the basis of the Newcastle-Ottawa Scale.
