# Supplementary material for: The Incidence of Epstein-Barr Virus-Positive Diffuse Large B-Cell Lymphoma: A Systematic Review and Meta-Analysis
Source: Cancers (Basel). 2021 Apr 8;13(8):1785. doi: 10.3390/cancers13081785 (PMC8068359; doi:10.3390/cancers13081785)
Supplement: Supplementary file 1 [file cancers-13-01785-s001.pdf]

Table S1. Quality ratings for the included studies on the basis of the Newcastle-Ottawa Scale.

Reference	Design	Selection	Comparability	Outcome or Exposure*	Total Score
Ahn JS et al. 2013	Cohort	4	2	3	9
Aladily TN et al. 2019	Cohort	4	0	1	5
Beltran BE et al. 2018	Cohort	4	2	3	9
Carreras J et al. 2017	Cohort	4	2	3	9
Chang ST et al. 2014	Cohort	4	2	3	9
Cohen M et al. 2017	Cohort	4	2	2	8
Gibson SE et al. 2009	Cohort	4	0	1	5
Hong JY et al. 2015	Secondary analysis	4	2	3	9
Hsueh CY et al. 2019	Cohort	4	2	3	9
Hu LY et al. 2017	Cohort	4	2	3	9
Keane C et al. 2019	Cohort	4	2	3	9
Lu TX et al. 2015	Cohort	4	2	3	9
Miyagi S et al. 2020	Cohort	4	2	3	9
Monabati A et al. 2016	Cohort	4	2	1	7
Naeini YB et al. 2016	Cohort	3	0	1	4
Ohashi A et al. 2017	Cohort	4	2	3	9
Ok CY et al. 2014	Cohort	4	2	3	9
Okamoto A et al. 2017	Cohort	4	2	3	9
Ozsan N et al. 2013	Cohort	4	0	2	6
Pan Y et al. 2013	Cohort	4	0	2	6
Salas MQ et al. 2020	Cohort	4	2	3	9
Sato A et al. 2014	Cohort	4	2	3	9
Slack GW et al. 2014	Cohort	4	2	3	9
Song CG et al. 2015	Matched case control study	4	2	3	9
Stuhlmann-Laeisz C et al. 2016	Cohort	4	0	3	7
Tokuyama K et al. 2017	Cohort	4	2	3	9
Uccini S et al. 2015	Cohort	4	0	3	7
Uner A et al. 2011	Cohort	4	0	2	6
Wada N et al. 2011	Cohort	4	0	2	6
Xie Y et al. 2014	Cohort	4	0	3	7
Ziarkiewicz M et al. 2016	Cohort	4	2	3	9

Note: rating of study quality: 8–9, very good; 6–7, good; 4–5, satisfactory; 0–3, unsatisfactory.

*For a case-control study by Song CG et al., the “Exposure” domain was evaluated. Otherwise, the “Outcome” domain was evaluated.
